# Weekly paclitaxel and cisplatin as neoadjuvant chemotherapy with locally advanced breast cancer: a prospective, single arm, phase II study

**DOI:** 10.18632/oncotarget.17954

**Published:** 2017-05-17

**Authors:** Liheng Zhou, Shuguang Xu, Wenjin Yin, Yanpin Lin, Yueyao Du, Yiwei Jiang, Yaohui Wang, Jie Zhang, Ziping Wu, Jinsong Lu

**Affiliations:** ^1^ Department of Breast Surgery, Renji Hospital, School of Medicine, Shanghai Jiaotong University, Shanghai, China; ^2^ Department of Breast Surgery, Fudan University Shanghai Cancer Center, Shanghai, China

**Keywords:** cisplatin, paclitaxel, breast cancer, neoadjuvant, biomarker

## Abstract

There was little evidence of weekly cisplatin regimen either for the locally advanced breast cancer or the metastatic setting. We aimed to evaluate that whether the combination of weekly paclitaxel and cisplatin could improve the efficacy of the neoadjuvant treatment for patients with locally advanced breast cancer. Patients with histologically confirmed large operable breast cancer received paclitaxel 80mg/m^2^ by weekly for 16 weeks and weekly cisplatin 25mg/m^2^ on day 1, 8 and 15, out of every 28 days for 4-week cycles. Trastuzumab was allowed for HER2-positive disease as weekly continuous regimen. The primary endpoint was locoregional total pathological complete response (tpCR) in breast and axilla lymph nodes after neoadjuvant treatment. One hundred and thirty-one patients were included in the study, among which 34.4% (45/131) patients achieved tpCR. Rate of pathological complete response (pCR) in the breast was 44.3% and the rate of near-pCR in breast was 48.1%. A significantly higher proportion of tpCR was seen in patients with triple negative breast cancer (64.7%, *p =* 0.003) and HER2 positive (non-luminal) cancer (52.4%, *p =* 0.018) compared with those who had luminal type tumors (24.7%). At multivariate analysis, negative estrogen receptor and high ki67 level independently predicted a better response. The most frequent toxicities were anemia, leukopenia and peripheral sensory neuropathy. Neoadjuvant chemotherapy by weekly paclitaxel and cisplatin combination was highly effective and tolerated in this study, especially in the triple negative and HER2 positive tumors**.**

## INTRODUCTION

Neoadjuvant chemotherapy is a guideline-based treatment for locally advanced breast cancer (LABC). It may decrease the tumor size and reduce clinical stage to facilitate surgical treatment. However, some chemotherapy regimens commonly used in adjuvant treatment phase did not show good efficacy for neoadjuvant purpose. Studies suggested that paclitaxel, either weekly or tri-weekly regimen, followed by FAC achieved pCR between 6%-19% in patients with luminal breast cancers and 30% for estrogen receptor (ER) negative and HER2 negative tumors [1]. Patients with operable IA-IIIA breast cancer had pCR by 26.1% by giving AC followed by with docetaxel in NSABP B27 [2]. In untreated females with different staged breast cancers, the pCR was 20.5% despite a combination chemotherapy of docetaxel, doxorubicin and cyclophosphamide in GeparTrio study [3]. More recently, a meta-analysis including 11955 patients from 12 multicenter neoadjuvant trials confirmed that pCR was significantly associated with event-free survival and overall survival [4]. Therefore, it is clinically important to explore a higher effective chemotherapy regimen to improve pCR in LABC including triple negative breast cancer (TNBC) as well as other various subtypes.

A randomized controlled study from M.D. Anderson Cancer Center indicated that a switch of i.v. paclitaxel from once every 3 weeks to weekly administration significantly improved the eradication of invasive cancer cells in the breast and lymph nodes [5].On the other hand, cisplatin-based neoadjuvant chemotherapy was explored to induce good clinical response in LABC, especially the TNBC [6-9]. Frasci [10] reported a phase 2 study showed a high pCR rate (62.0%) in TNBC after the administration of weekly cisplatin-based chemotherapy (cisplatin, epirubicin, paclitaxel). This weekly regimen of cisplatin-epirubicin-paclitaxel further improved both distant metastasis-free survival and overall survival compared with the tri-weekly combination of epirubicin and paclitaxel in SICOG 9908 trial [11]. The SWOG S0221 trial showed that once-per-week doxorubicin-cyclophosphamide did not improve the DFS as the adjuvant therapy when compared with the once every 2-weeks regimens [12].

Based on the above evidence, we hypothesized that a non-anthracycline-containing regimen by a combination of platinum and taxane may be effective in patients with LABC by improving the pathological complete remission in various biological subtypes. The efficacy of taxanes and platinum may exert via the mechanisms of metronomic chemotherapy or regulation of antitumor immune responses. The aim of this study was to assess efficacy and safety of a specific neoadjuvant regimen by weekly i.v. paclitaxel and cisplatin in patients with LABC.

## RESULTS

### Patient characteristics

Between January 2013 and June 2015, 132 women were enrolled in the study. One woman had no surgery and was lost to follow-up, and one had delayed operation due to serious rheumatoid arthritis and pneumonia. In total, 131 patients were eligible for response evaluation and included in the safety and efficacy analysis. The median age was 49 years (range 23-70 years). Sixty-five patients (49.6%) were postmenopausal, and 66 (50.4%) were premenopausal, among them two had hysterectomy. In terms of hormonal receptor, ki67 and HER2 statuses, according to the St Gallen international expert consensus [13], there were 8 (6.1%) patients with Luminal A-like breast cancer, 85 (64.9%) were Luminal B-like, among them 32 were HER2 positive and 53 were HER2 negative. Twenty-one (16%) patients were HER2-positive (non-luminal) and 17 (13%) were triple-negative. The median tumor size of the entire group was 6 cm at baseline and 58.1% tumors were T3 or T4. Table1 lists the baseline patient’s characteristics.

**Table 1:** Characteristics of patients and the tumors at baseline

Characteristic	Number of patients	%
Age-years		
≤35	15	11.5
35∼50	55	42
>50	61	46.6
Menopausal status		
Premenopausal	66	50.4
Postmenopausal	65	49.6
Primary tumor size		
T1, ≤2cm	10	7.6
T2, 2-5cm	45	34.4
T3-4, >5cm	76	58
Nodal status		
N0	14	10.7
N1-3	117	89.3
Tumor stage		
IIA	9	6.9
IIB	43	32.8
IIIA	70	53.4
IIIB	4	3.1
IIIC	5	3.8
ER		
Positive	85	64.9
Negative	46	35.1
PR		
Positive	84	64.1
Negative	47	35.9
HER2 status		
Positive	53	40.5
Negative	78	59.5
Molecular classification		
Luminal A-like	8	6.1
Luminal B-like (HER2 negative)	53	40.5
Luminal B-like (HER2 positive)	32	24.4
HER2 positive (non luminal)	21	16
Triple negative	17	13

ER, estrogen receptor; PR, progesterone receptor; HER2, human epidermal growth factor receptor 2.

One hundred and sixteen patients completed all the four cycles of chemotherapy, and 15 patients only received three cycles (one developed rash due to allergic to the peripherally inserted central catheter; other 14 refused to continue). The proportion of treatment discontinuations was 11.5%. There were 31 patients had dose modifications resulting from toxicities. Among 53 patients with HER2 over-expression breast cancer, including Luminal B-like (HER2 positive) and HER2 positve (non-luminal) subtypes, 38 patients had trastuzumab for both neoadjuvant and adjuvant setting. Another 3 patients only had trastuzumab for adjuvant treatment. All patients then received modified radical mastectomy. One patient underwent immediate breast reconstruction with latissimus dorsi muscular flap and implant.

### Efficacy

**Figure 1:**
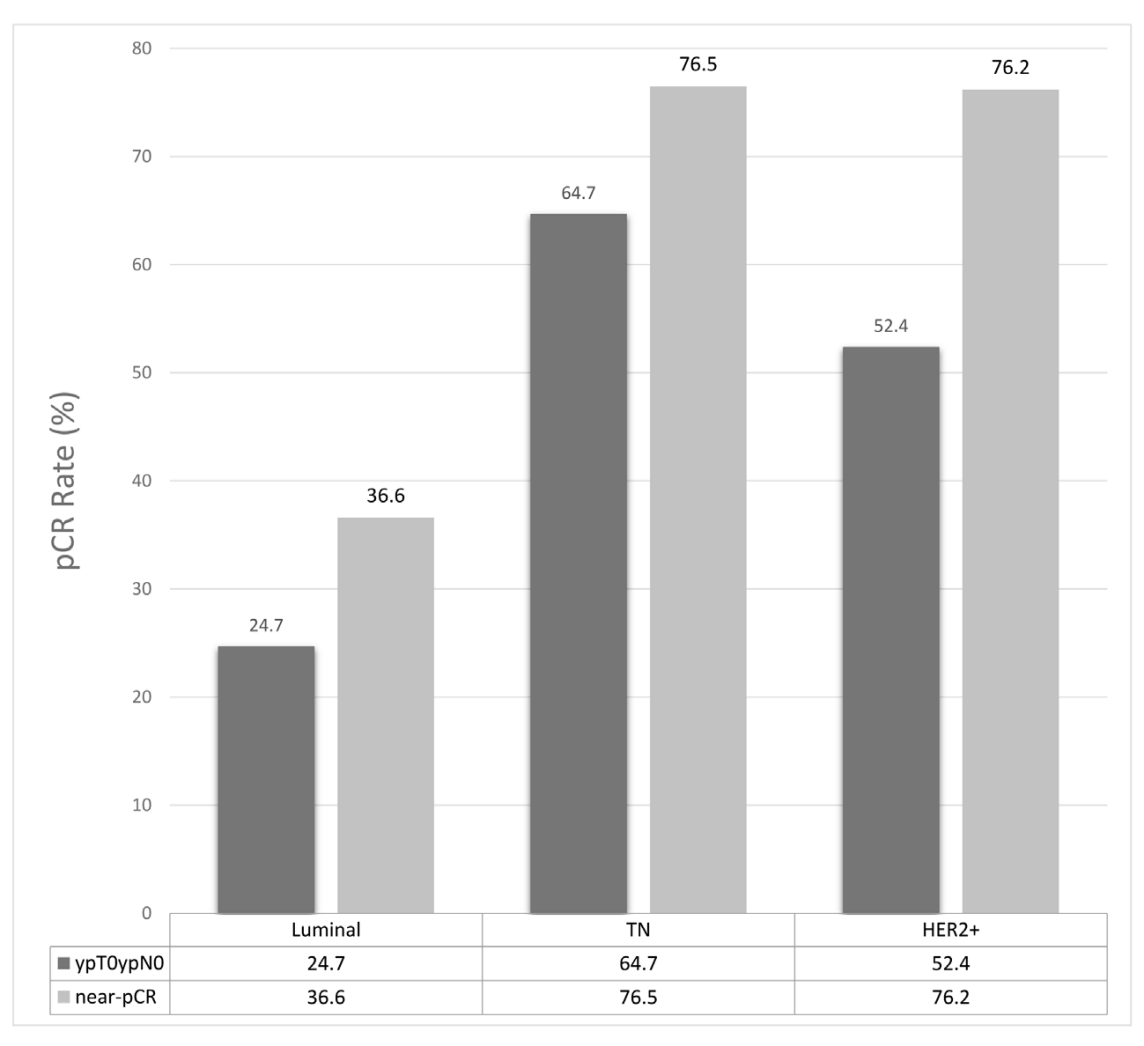
Pathological response according to different biomarkers Luminal: Luminal A-like and Luminal B-like (HER2 negative and HER2 positive); TN: Triple negative; HER2+: HER2 positive (non-luminal).

**Figure 2:**
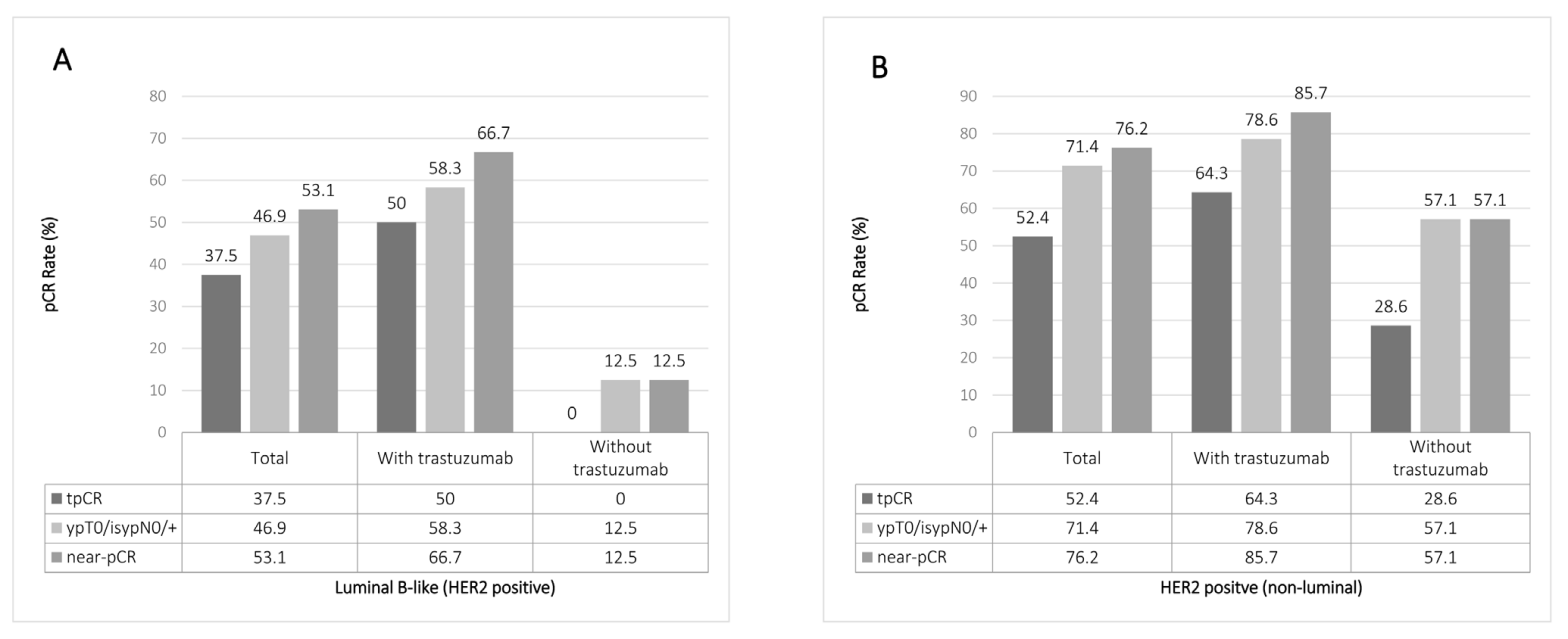
Pathological response according to trastuzumab treatment in HER2 overexpression patients Analysis of correlation of trastuzumab treatment and pathological complete response in patients with Luminal B-like (HER2 positive) breast cancer (**A**) or HER2 positive (non-luminal) (**B**) tumors.

We observed 19 tpCR among 54 patients in the first stage and continued to the second stage with 78 additional patients. Further 26 complete responses were seen in the second stage, resulting in a tpCR rate of 34.4% for the entire group (Table 2). Besides, 44.3% of patients achieved pCR in breast and 48.1% achieved near-pCR in breast. An analysis of response rate in the breast and lymph nodes based on molecular classification is showed in figure 1. Patients with triple negative tumors (tpCR 64.7%, *p =* 0.003), as well as those with HER2-positive (non-luminal) tumors (tpCR 54.2%, *p =* 0.018) had higher tpCR rates compared with those Luminal tumors (tpCR 24.7%). Among the 53 patients with HER2 over-expression tumors, 16 of 24 (66.7%) patients who had Luminal B-like (HER2 positive) tumors achieved a significantly higher near-pCR rate with trastuzumab compared with 1 of 8 (12.5%) without trastuzumab (*p =* 0.024) (Fig 2). In the HER2 positive (non-luminal) tumors, 12 of 14 (85.7%) patients with trastuzumab achieved a near-pCR compared with 4 of 7 (57.1%) without trastuzumab, but the difference was not significant (*p =* 0.223). In this trial, no patients had disease progress and the overall clinical response rate was 92.4%. The mean tumor size of the 63 patients with no residue invasive cancers in breast was 5.6cm. Twenty-six of 76 patients (34.2%) with T3 or T4 tumors achieved tpCR.

**Table 2:** Pathological response rates at time of surgery

Pathological response	Number of patients	%
Complete response		
tpCR(ypT0ypN0)	45	34.4
pCR in breast (ypT0/isypN0/+)	58	44.3
near-pCR in breast	63	48.1
Partial response	58	44.3
Stable Disease	10	7.7
Progression disease	0	0

ypT0ypN0: pathological response rate in breast and axillary lymph node

ypT0/isypN0/+: absence of invasive tumor cells in the breast

near-pCR: only a few scattered tumor cells remained or that the residual tumor was <0.5cm in size in the breast

### Predictive markers

Univariate analyses showed that negative ER and PR status, high ki67 expression was significantly associated with high pCR rate (p < 0.05). Dose reduction of chemotherapy (*p =* 0.666) or failure to complete all the cycles (*p =* 0.577) was not significantly associated with the pCR (Table 3). Multivariate logistic regression analysis indicated that ER (OR = 0.294, 95%CI 0.09∼0.957, *p =* 0.042) and ki67 status (OR = 7.852, 95%CI 1.686∼36.558, *p =* 0.009) were independent predictors (Table 4) to tpCR. In this study, the baseline tumor size was not a predictor for tpCR (≤5 cm vs.>5 cm, OR = 0.59, 95%CI 0.229-1.522, *p =* 0.275). Treatment of trastuzumab also significantly increased the tpCR rate (*p =* 0.049).

**Table 3:** Comparison of treatment efficacy by various factors

Variable	No. of Patients (%)		*P*
	pCR	Non-pCR	
Age			
<35	7(15.6)	8(9.3)	0.525
35∼50	19(42.2)	36(41.9)	
>50	19(42.2)	42(48.8)	
Tumor size			
<5cm	19(42.2)	36(41.9)	1.000
≥5cm	26(57.8)	50(58.1)	
Lymph Node			
Negative	6(13.3)	8(9.3)	0.555
Positive	39(86.7)	78(90.7)	
Postmenopausal			
Yes	18(40)	47(54.7)	0.141
No	27(60)	39(45.3)	
ER status			
Negative	26(57.8)	20(23.3)	0.000
Positive	19(42.2)	66(76.7)	
PR status			
Negative	26(57.8)	21(24.4)	0.000
Positive	19(42.2)	65(75.6)	
Ki67			
<20%	3(6.8)	27(32.1)	0.001
≥20%	41(93.2)	57(67.9)	
HER2 status			
Negative	23(51.1)	30(34.9)	0.092
Positive	22(48.9)	56(65.1)	
Dose reduction			
Yes	12(26.7)	19(22.1)	0.666
No	33(73.3)	67(77.9)	
Complete full cycles			
Yes	41(91.1)	75(87.2)	0.577
No	4(8.9)	11(12.8)	

ER, estrogen receptor; PR, progesterone receptor; HER2, human epidermal growth factor receptor 2; pCR, pathologic complete response.

**Table 4:** Multivariate analysis of the predictive markers of tpCR

Variable	Comparison for Risk Ratio	Risk Ratio	95% Confidence Interval	*P*
Age	≤50 *versus* >50	1.909	0.433-8.411	0.393
Tumor size	≤5 cm *versus* >5 cm	0.59	0.229-1.522	0.275
Clinical lymph node status	Negative *versus* positive	0.339	0.081-1.421	0.139
Trastuzumab	With *versus* without	0.161	0.026-0.989	0.049*
Menopausal status	Post *versus* Pre	3.001	0.657-3.702	0.156
ER status	Negative *versus* positive	0.294	0.09-0.957	0.042*
PR status	Negative *versus* positive	0.46	0.145-1.462	0.188
Ki67	Low *versus* high	7.852	1.686-6.558	0.009*
HER2 status	Positive *versus* negative	1.975	0.311-2.524	0.47

ER, estrogen receptor; PR, progesterone receptor; HER2, human epidermal growth factor receptor 2.

* Significant value.

### Toxicity

The hematological toxicities were described in all 131 analyzed patients completed neoadjuvant treatment. The most common adverse event reported was anemia, but patients had an acceptable level of grade 2 (34, 26.2%) or grade 3 (3, 2.3%) anemia. The incidence of patients with grade 3 or 4 neutropenia was 37.4%, who were resolved using G-CSF. Only 2 patients (1.5%) had transient slight increase in creatinine level who had clinical resolution after the treatment. Non-hematological events were estimated in 85 patients. Nausea occurred commonly, with the frequency of grade 1 and 2 events at 50.6%. Peripheral sensory neuropathy was frequent (65.9%) but never severe. Only one patient who was hospitalized due to probable allergy to paclitaxel had a serious adverse event. No significant cardio-toxicity was observed in any patient, especially in patients treated with Trastuzumab. No deaths were associated with the treatments in this study. Toxicities of all the patients are listed in Table 5.

**Table 5:** Frequency of Hematological (*n* = 131) and non-hematological toxicity (*n* = 85)

		Grades 1-2	Grade 3		Grade 4
Haematological toxicity					
Anemia	88(67.2)		3(2.3)	0	
Leukopenia	79(60.3)		24(18.3)	1(0.8)	
Neutropenia	51(38.9)		31(23.7)	18(13.7)	
Alanine aminotransferase increased(ALT)	24(18.3)		0	0	
Alanine aminotransferase increased (AST)	27(20.6)		0	0	
Creatinine increased	2(1.5)		0	0	
Non-haematological toxicity					
Nausea	43(50.6)		6(7.1)	0	
Vomiting	21(24.7)		2(2.3)	0	
Diarrhea	36(42.3)		2(2.3)	0	
Constipation	30(35.3)		0	0	
Fever	18(21.2)		1(1.2)	0	
Hand-foot syndrome	18(21.2)		0	0	
Peripheral neuropathy	56(65.9)		0	0	
Skin rash	20(23.5)		0	0	
Epistaxis	10(11.8)		0	0	

Data are *n* (%)

## DISCUSSION

As far as we know, we first reported the response and safety results of non-anthracycline containing weekly paclitaxel and cisplatin neoadjuvant chemotherapy in patients with LABC. The data reported for the first time herein suggested that weekly paclitaxel and cisplatin for a total of 16 weeks resulted into a high pCR rate in large operable breast cancer and were well tolerated. Higher proportions of patients who achieved pCR were seen in triple negative and HER2 positive breast cancers.

Cisplatin is a chemotherapeutic agent not used routinely for breast cancer yet, but studies increasingly showed its good response in subsets of breast cancer. Triweekly cycles of different cisplatin based neoadjuvant chemotherapy regimens showed similar efficacy in LABC patients. A study by Silver et al. on 28 patients with TNBC showed a pCR of 22% by four cycles of single cisplatin at 75 mg/m^2^ at every 21 days [6]. Villman [8] reported a similar efficacy (pCR 19%) of 3-weekly cycles of neoadjuvant epirubicin, cisplatin and capecitabine in LABC. Another small scale trial showed that 14.3% of LABC patients achieved pCR by the combination of tri-weekly docetaxel, capecitabine and cisplatin [14]. The sequential use of doxorubicin and cisplatin/docetaxel every 3 weeks as neoadjuvant therapy also showed a pCR rate of 24% in LABC [15]. Up to now, there was no reports for the weekly paclitaxel combined with weekly cisplatin in the neoadjuvant setting. In this study, we showed a higher activity of this anthracycline-free regimen, while the tpCR rate was 34.4%, the rate of pCR in the breast was 44.3% and the near-pCR rate in the breast was 48.1%. Furthermore, there was no patient who had disease progressed. In fact, baseline patients’ characteristics were not clinically favorable in terms of breast cancer staging. The mean tumor size was 6 cm, more than half of patients had T3 or T4 disease and 89.3% had clinically positive axilla. This treatment protocol provided inspiring treatment results with a 92.4% clinical response rate according to the RECIST criteria after 4 cycles’ treatment.

The weekly cisplatin group was not directly compared with the triweekly cisplatin before. Which platinum salt is the best to be added to neoadjuvant is still unknown. In the TBCRC009 trial, however, the results showed the objective response rate was numerically higher with cisplatin than with carboplatin for the patients with metastatic TNBC received cisplatin or carboplatin once every 3 weeks [16]. We hypothesized that administration of cisplatin on a more frequent schedule may improve the pathophysiologic responses in patients with locally advanced disease. According to the GeparSixto trial, the pCR rate was 43.7% for the patients with triple-negative breast cancer in the weekly carboplatin group [4]. CALGB 40603 study, designed to have 3-weekly dosing of carboplatin, showed that addition of carboplatin significantly increased the pCR rate to 54% [17]. According to our result, the rate of pCR and near-pCR were 64.7% and 76.5% in the TN patients and were 52.4% and 76.2% in the HER2-positive patients, which was numerically higher than the previous reported studies. Our study indicated that weekly schedule of cisplatin chemotherapy was highly effective as neoadjuvant chemotherapy.

A meta-analysis suggests there is higher hematologic toxicity rates by carboplatin treatment in the first-line treatment [18]. Nephrotoxicity is the major dose-limiting effect of cisplatin and therefore, either continuous hydration for over 24h or short hydration regimen is recommended after cisplatin administration. However, in our trial the weekly use of low dose of cisplatin was well tolerated without continuous hydration. This study showed the proportion of major toxicity of reversible anemia was 69.5%. This trial also showed that the dose reduction of cisplatin and paclitaxel may reduce the incidence of adverse events and had no detrimental effect on efficacy as well. So the optimum dose of cisplatin and paclitaxel needs to be established in future studies.

A major limitation of available data is the lack of long-term prognosis. The clinical trial is still on going and five-year follow-up is scheduled. Although we didn’t have data to directly compare the weekly cisplatin schedule with the 3-weekly cycles of cisplatin or carboplatin, our results indicated that weekly cisplatin was an effective therapeutic alternative for LABC. Otherwise, Borski’s report of a neoadjuvant trial using the 3 weeks cisplatin regimen in women with germline Breast Cancer 1 (BRCA1) mutations suggested that tumors from women with hereditary BRCA1 mutations had a high rate of response to cisplatin [19]. Cisplatin-based chemotherapy was seemed to be effective in a high proportion of patients with BRCA1 mutation breast cancers. However, this work is in progress in this study.

In conclusion, our herein study confirmed the combination neoadjuvant chemotherapy with weekly paclitaxel and weekly cisplatin was a very active and particularly well tolerated treatment for patients with LABC. However, we still need to pay attention to the relationship between the pCR rate and prognosis, so a long time follow up and a further large scale randomized study are required.

## PATIENTS AND METHODS

### Eligibility criteria

Patients were eligible if they were between 18 and 70 years of age, had histologically confirmed and untreated large operable breast cancer (T size ≥2 cm and N0-2), had Eastern Cooperative Oncology Group (ECOG) performance status < 2, had adequate hematologic (granulocyte count ≥1.5×10^9^/L, platelet count ≥100×10^9^/L, hemoglobin level ≥90g/L) and hepatic (transaminases ≤1.5× the upper limit of normal [ULN], and bilirubin≤1.5UNL) functions, and had no major organ dysfunctions.

Patients were excluded if they were pregnant, had metastatic breast cancer, or had documented history of medical conditions that indicate intolerance to neoadjuvant therapy (uncontrolled cardiovascular disease or severe infection). Also excluded were patients who had a previous history of malignancy other than breast cancer or had received any prior radiation, chemotherapy, or hormonal therapy for their present breast cancer.

Patients were screened in a clinic or an inpatient department, and were hospitalized once included. Written informed consent was obtained from all patients before screening. The study was conducted in accordance with the guidelines of the International Conference on Harmonization, and was approved by independent ethics committees of two study sites, RenJi Hospital, Shanghai Jiao Tong University and Fudan University Shanghai Cancer Center. This study was registered with ClinicalTrials.gov. as NCT02199418.

### Study design and treatment

**Figure 3:**
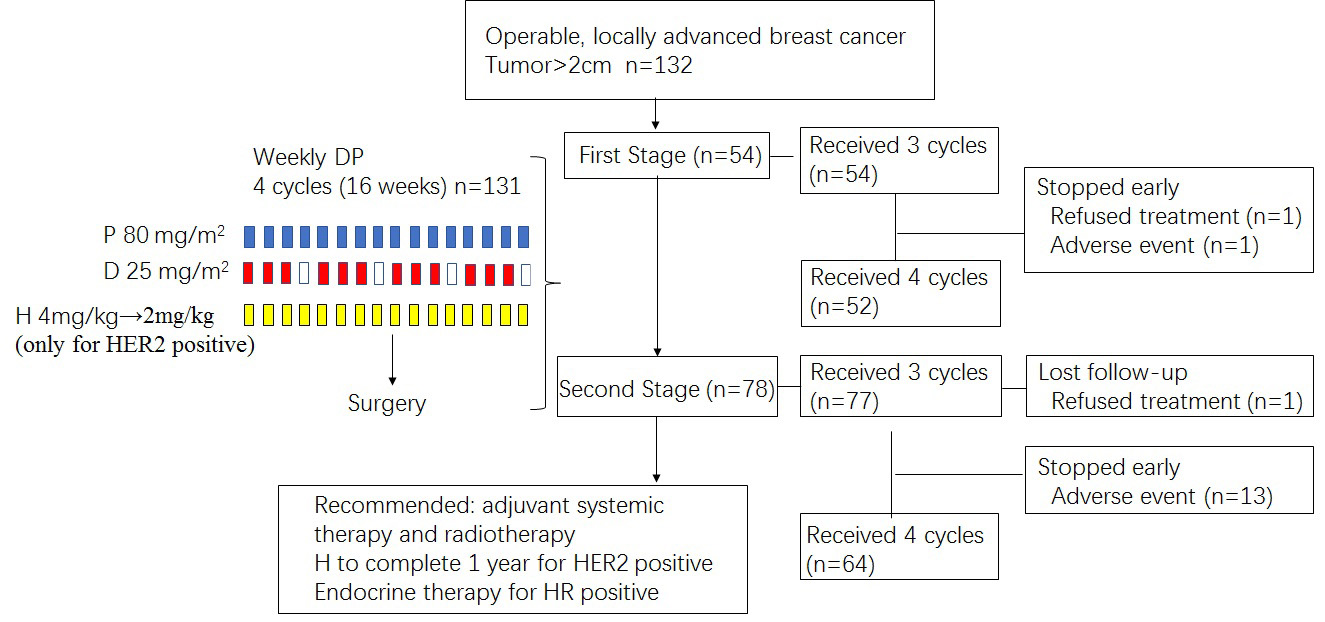
Treatment schema P, paclitaxel; D, cisplatin; H, trastuzumab; HR, hormonal receptor.

This study was designed as an open-label, prospective, single-arm, phase 2 clinical trial, assessing the efficacy and safety of neoadjuvant chemotherapy by a combination of weekly paclitaxel and cisplatin. The treatment plan is illustrated in Figure 3. Patients were scheduled to receive i.v. infusion of paclitaxel 80mg/m^2^ weekly on day 1 for 16 weeks and cisplatin 25mg/m^2^ on day 1, 8, and 15 every 28 days for 4 cycles. Patients were given dexamethasone 20 mg intravenously before the start of the first cycle and 10mg before each following cycle. Patients with HER2-positive cancer were allowed to use concomitant trastuzumab at a weekly basis. The first dose of trastuzumab was 4mg per kg of body weight and the subsequent doses were 2 mg per kg. Granulocyte colony-stimulating factor (G-CSF) administration was also allowed if deemed necessary.

Chemotherapy was delivered at full doses if absolute neutrophil count (ANC)>1.5 ×10^9^/L and platelets>100×10^9^/L. In presence of lower ANC, higher transaminases, or other serious adverse events, dose adjustment was made to reduce by 10%∼25% of the full doses and was permitted to delay or omit according to discretions of the study physicians.

Baseline evaluation include a complete medical history and physical examination, electrocardiogram, chest computed tomography scan, abdominal ultrasound, contralateral mammography and breast magnetic resonance imaging. A core biopsy of the tumor in the breast was also carried out with the immunohistochemical assessment of the biomarkers (steroid hormone receptors, HER-2 and ki-67). ER/ progesterone receptor (PR) positive is defined as ≥1% stained cells and HER2-positive is defined as immuno-histochemistry 3+ or the ratio of HER2 gene signals to chromosome 17 signals >2.0 or HER2 gene copy >6.0.

Additional two cycles of weekly paclitaxel and cisplatin or four cycles of cyclophosphamide, epirubicin and fluorouracil (CEF) were recommended as the adjuvant chemotherapy. Radiotherapy was delivered according to the radiologist. Trastuzumab for 1 year in total was recommended for patients with HER2 positive tumors. Adjuvant endocrine therapy was also recommended for patients with HR positive tumors.

### Endpoints

The tpCR was the primary endpoint of the study, which defined as the absence of tumor in the breast and axillary lymph nodes sample taken at the time of surgery. The rate of pCR only in the breast, with absence of invasive tumor cells in the breast, and the rate of near-pCR in the breast which meant that only a few scattered tumor cells remained or that the residual tumor was < 0.5cm in size [20] were also estimated as exploratory post-hoc analysis. The secondary outcome measures included clinical response, tolerability and safety.

Toxicity was assessed at each visit and recorded according to the National Cancer Institute-Common Toxicity Criteria version 3.0. Complete blood cell counts were performed twice a week at each cycle and chemistry performed once every cycle for all patients.

### Statistical analysis

Simon’s two-stage design [21] was used to estimate number of patients. The null hypothesis that the true response rate was 25% would be tested against a one-sided alternative. In the first stage, 54 patients would be accrued. If there were 13 or fewer responses in these patients, the study will be stopped. Otherwise, 78 additional patients would be accrued. This design yielded a type I error rate of 0.05 and power of 0.8 when the true response rate was 35%. Response was assessed according to Response Evaluation Criteria In Solid Tumors (RECIST) 1.1. Number of participants with treatment-related adverse events was summarized with descriptive statistics. Patients with different responses were compared with the use of a continuity-corrected two-sided Pearson’s chi-squared test or Fisher’s exact test. A post-hoc logistic regression analysis was used to explore factors predictive of tpCR. All statistical analyses were done with SPSS Statistics software (version 22) and a p-value of 0.05 was considered statistically significant unless otherwise specified.

## Abbreviations

LABC: locally advanced breast cancer
i.v: intravenous
tpCR: total pathological complete response
pCR: pathological complete response
TNBC: triple negative breast cancer
HER2: human epidermal growth factor receptor 2
ER: estrogen receptor
ULN: upper limit of normal
G-CSF: granulocyte colony-stimulating factor
PR: progesterone receptor
RECIST: Response Evaluation Criteria in Solid Tumors
BRCA1: Breast Cancer 1
